# Possible contribution of COVID-19 vaccination to the subsequent mental well-being in Japan

**DOI:** 10.1038/s41598-022-25357-1

**Published:** 2022-12-07

**Authors:** Chifa Chiang, Shuhei Morita, Yoshihisa Hirakawa, Farzana Tanzin Priya, Yuka Matsumoto, Atsuhiko Ota, Hiroshi Yatsuya, Takahiro Tabuchi

**Affiliations:** 1 Department of Public Health and Health Systems, Nagoya University Graduate School of Medicine, 65 Tsurumai-Cho, Showa-ku, Nagoya, Aichi 466-8550 Japan; 2 Nagoya University School of Medicine, 65 Tsurumai-Cho, Showa-ku, Nagoya, Aichi 466-8550 Japan; 3 Department of Public Health, Fujita Health University School of Medicine, Toyoake, Aichi 470-1192 Japan; 4 Cancer Control Center, Osaka International Cancer Institute, 3-1-69 Otemae, Chuo-ku, Osaka-shi, Osaka 541-8567 Japan

**Keywords:** Public health, Risk factors

## Abstract

The coronavirus disease 2019 (COVID-19) pandemic has had a severe impact on mental well-being. Vaccination may have played a pivotal role in enduring this mental health crisis. The present study aimed to longitudinally investigate the association between COVID-19 vaccination and mental health status among Japanese population in 2021. Longitudinal data of 17,089 individuals aged 15–79 years who participated in a nationwide online study were analyzed. Baseline and follow-up mental health statuses were assessed using the Kessler Psychological Distress Scale (K6). General linear and multivariable logistic regression models adjusted for baseline levels of mental distress were used to examine the association between vaccine receipt and follow-up levels of mental health. Mean K6 scores were lower in the vaccinated than in the non-vaccinated participants. Those who had received one or two doses of COVID-19 vaccines were associated with improved mental health at follow-up in subjects with psychological distress at baseline (odds ratio [OR] 1.31 and 1.35, respectively) and were inversely associated with deteriorated mental health status at follow-up in subjects without psychological distress at baseline (OR 0.66 and 0.70, respectively) compared with no vaccination groups, respectively. The present study would indicate that one or two doses of COVID-19 vaccinations contributed to mental well-being in Japan. This finding might provide evidence for promoting vaccination against COVID-19 and emerging infectious diseases in the future.

## Introduction

At the initial stage of the coronavirus disease 2019 (COVID-19) pandemic, many countries had imposed strict restrictions on gatherings and movement to contain the spread of the virus. To mitigate the impact on the domestic economy, governments were forced to adjust their virus control strategies repeatedly. A safe and effective vaccine against the severe acute respiratory syndrome coronavirus 2 (SARS-CoV-2) is pivotal to a possible exit strategy from the crisis.

The Japanese government specially approved the use of the Pfizer-BioNTech COVID-19 Vaccine (also known as Comirnaty) on February 14th, 2021, as the first authorized vaccine against SARS-CoV-2 in Japan, and launched its vaccination on February 17th, 2021, first targeting front-line medical professionals. As of early August 2021, approximately 80% of the older people aged 65 years or over had received two doses of vaccines. Moreover, the government reported that approximately 77% of the entire Japanese population had been vaccinated with two doses by the end of November 2021^1^.

The pandemic had a severe impact on people’s mental well-being^2,3^. It was estimated that there was an alarming increase in depressive (27.6%) and anxiety (25.1%) disorders globally in 2020, including in Japan; daily SARS-CoV-2 infection rates and reductions in human mobility were reportedly two of the major contributors to this increase^4^. As of now, there is still little being reported on the global mental health status in 2021, the second year of the lasting pandemic. However, a sign of gradual recovery from the predicament was observed. A nationwide online survey conducted by Japan’s Ministry of Health, Labour and Welfare, targeting residents aged ≥ 15 years in late November 2021, reported that the number of people suffering from nervousness had decreased from 21.7% in April-June 2021 to 12.4% in October–November 2021^5^. Those who felt anxiety about infections with SARS-CoV-2 in themselves or in family members had decreased from 62.8% in April-June 2021 to 42.4% in October–November 2021. It was speculated that the increased vaccination rate of the population had contributed to the decreased number of new infections or a lower percentage of severe cases among infected people, and thus the entire society had recovered from the COVID-19 recession gradually; people’s mental well-being had improved concurrently or consequently over the period.

Among the few published papers on this topic, a nationally representative cohort study carried out in the United States and a cross-sectional study carried out in the southern area of China have reported decreased mental distress levels after receiving the first dose of COVID-19 vaccines^6–8^. Although the linkage between the COVID-19 vaccination rate and improved mental health status of the Japanese population was observed in chronological order in 2021, the association between general individuals’ mental health improvement and COVID-19 vaccination has not yet been well examined in Japan. The present study aimed to longitudinally investigate the association between improvement in mental health status and (one- or two-dose) COVID-19 vaccination in 2021, after the vaccine became widely available in Japan.

## Results

A total of 17,089 participants (8692 males and 8397 females) with a mean age of 52.5 (standard deviation = 16.3) years were analyzed in the present study. The mean age of those who received two doses was higher than that of the one-dose vaccination or no vaccination groups (p < 0.001) (Table 1). The percentage of university graduates or above education was higher in those who received one or two doses of the vaccine than in those who did not receive any vaccines. The percentage of vaccine hesitancy, i.e., those who indicated that they did not want to get vaccinated, at baseline was much higher in the non-vaccinated group (35%) than the vaccinated groups (one-dose 12%, two-dose 6%). The percentage of being infected with COVID-19 in the past one year was lower in the two-dose vaccination than that in one-dose vaccination or no vaccination groups. As shown in Table 2, the adjusted mean Kessler Psychological Distress Scale (K6) score of the no vaccination group at follow-up was higher than that of the one- or two-dose vaccination groups (p < 0.001). No significant difference in the mean K6 scores was observed between the one- and two-dose vaccination groups. Consistent results were obtained from the analyses excluding participants with higher levels of baseline psychological distress (Supplementary Table S1).

**Table 1:** Characteristics of participants according to COVID-19 vaccination status, 2021, JACSIS, Japan (n = 17,089).

Variables	Vaccination status (number of doses received)			*p*
	None	One	Two	
Age, mean (SD), years	44.7 (14.9)	41.0 (12.4)	55.6 (15.8)	< 0.001
**Sex, %**				
Female	51.2	51.2	48.4	0.007
**Educational attainment, %**				
Junior high school	5.3	3.8	2.3	< 0.001
Senior high school	34.0	29.5	30.7	
2-year college graduate	22.8	20.0	20.4	
Bachelor's degree	34.3	41.7	42.2	
Graduate school degrees	3.6	5.0	4.5	
**Occupation, %**				
Healthcare workers	8.1	7.1	9.0	0.021
**Living arrangements, %**				
With family members	73.3	79.0	81.9	< 0.001
Frequency of outings at follow-up, mean (SD), day/month	14.2 (9.9)	15.1 (9.8)	16.0 (9.7)	< 0.001
Frequency of voice chatting at follow-up, mean (SD), day/month	8.3 (10.2)	8.9 (10.3)	10.0 (10.5)	< 0.001
FCV-19S scores at baseline, mean (SD)	17.2 (6.0)	17.7 (5.7)	18.3 (4.7)	< 0.001
FCV-19S scores at follow-up, mean (SD)	17.9 (5.5)	18.2 (4.9)	18.6 (4.8)	< 0.001
**Vaccine hesitancy at baseline, %**				
Yes	35.1	12.5	5.6	< 0.001
**COVID-19 infection, %**				
Yes	1.5	1.6	0.4	< 0.001

*COVID-19* coronavirus disease 2019, *SD* standard deviation, *FCV-19S* fear of COVID-19 scale.

**Table 2:** Mean K6 scores in total participants (n = 17,089) according to COVID-19 vaccination status, 2021, JACSIS, Japan.

	No vaccination	One-dose vaccination	Two-dose vaccination
n	2942	1417	12,730
**Mean K6 at baseline (SE)**			
Crude	5.75 (0.12)	6.00 (0.16)	4.31 (0.05)
Adjusted, Model 1	4.90 (0.10)	4.86 (0.15)	4.64 (0.05)
**Mean K6 at follow-up (SE)**			
Crude	5.37 (0.11)	4.96 (0.15)	3.54 (0.04)
Adjusted, model 1	4.61 (0.10)	3.94 (0.14)	3.83 (0.05)
Adjusted, model 2	4.46 (0.08)	3.85 (0.10)	3.87 (0.03)

Model 1: adjusted for sex, age, education, occupation, living arrangements.

Model 2: adjusted for sex, age, education, occupation, living arrangements, baseline K6, baseline vaccine hesitancy, frequency of outings at follow-up, frequency of voice chatting at follow-up, fear of COVID-19 scores at follow-up, and COVID-19 infection.

*K6* Kessler 6 scale, *COVID-19* coronavirus disease 2019, *SE* standard error.

**Figure 1:**
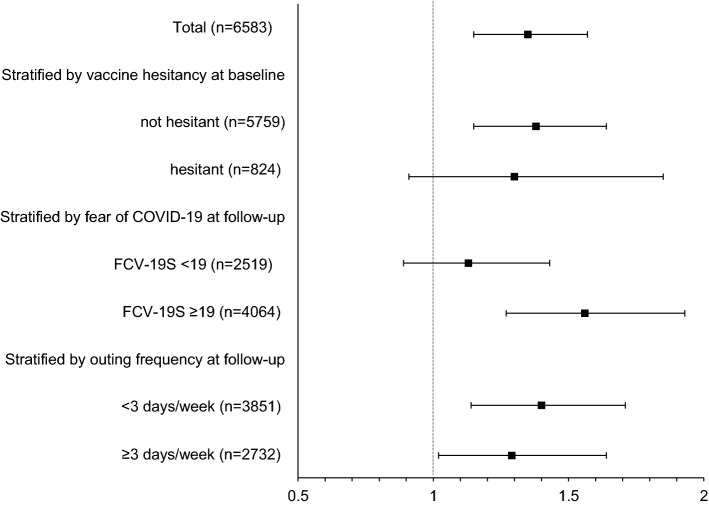
Adjusted odds ratios (two-dose vaccination vs. no vaccination) of improved mental health in participants with psychological distress at baseline, 2021, JACSIS, Japan.

**Figure 2:**
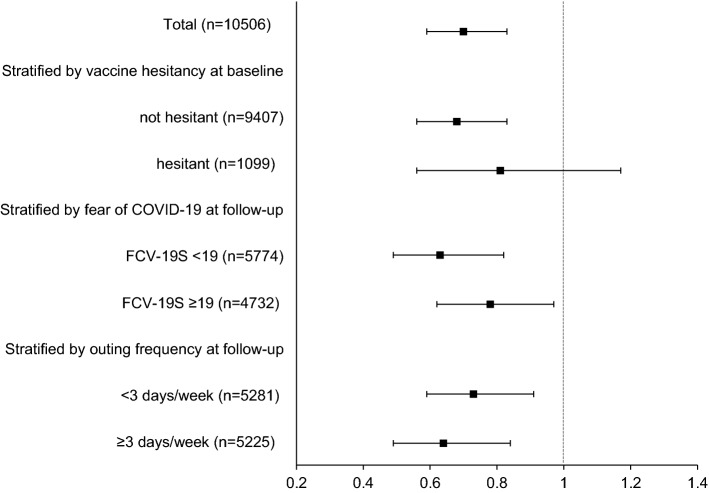
Adjusted odds ratios (two-dose vaccination vs. no vaccination) of deteriorated mental health in participants without psychological distress at baseline, 2021, JACSIS, Japan.

At baseline, 6583 subjects (39%) had psychological distress (K6 ≥ 5) and the remaining 10,506 (61%) did not. At follow-up, 5652 (33%) had psychological distress. Of the 6583 subjects with distress at baseline, 4289 (65%) continued having psychological distress at follow-up. Of 10,506 subjects without distress at baseline, 9143 (87%) remained free of distress at follow-up. The percentages of subjects with psychological distress at baseline were 46%, 48% and 36% in no, one-dose, and two-dose groups, respectively. Similarly, the percentages of subjects with psychological distress at follow-up were 44%, 41% and 30% in no, one-dose, and two-dose vaccination groups, respectively. Among the subjects with psychological distress at baseline, 27%, 32% and 38% of the subjects in no, one-dose, and two-dose vaccination groups, respectively experienced improvement of the psychological distress status (Supplementary Table S2). Based on the multivariable logistic regression models adjusted for baseline K6 scores and other potential confounding factors, the odds of improved mental health status were significantly higher in the one-dose (OR: 1.31, 95% CI 1.05–1.63) and two-dose vaccination (OR: 1.35, 95% CI 1.15–1.57) compared with that in the no vaccination group (Fig. 1). Among the subjects without psychological distress at baseline, 20%, 16%, and 11% experienced deterioration of the psychological distress status (Supplementary Table S3). Namely, the odds of deteriorated mental health status were significantly lower in the one-dose (OR: 0.66, 95% CI 0.51–0.85) and two-dose vaccination (OR: 0.70, 95% CI 0.59–0.83) compared with that in the non-vaccinated group (Fig. 2). Consistent results were obtained from sensitivity analyses adopting different K6 cutoffs (9 or 13 points instead of 5 points) for the outcome variable (data not shown).

Stratified analyses by age, vaccine hesitancy, fear of COVID-19 at baseline, or frequency of outing at follow-up did not indicate material differences according to the strata (Supplementary Tables S2 and S3).

## Discussion

To the best of our knowledge, the present study is the first to investigate the possible contributions of COVID-19 vaccination to mental health status using nationwide longitudinal data from the Japanese population. Our study revealed that mental health status assessed using the K6 scale was better in one-dose or two-dose vaccinated participants than in non-vaccinated ones even after adjustment for baseline K6 scores and vaccine hesitancy among the Japanese population aged 15–79 years.

The findings of the present study were consistent with previous studies carried out among the adult American population^6,7^ and regional Chinese residents^8^ reporting decreased mental distress levels after receiving the first dose of COVID-19 vaccines, although mental distress levels were assessed using the Patient Health Questionnaire (PHQ) in those previous studies rather than the K6 scale used in our study. In contrast, a Japanese study conducted in late June 2021 targeting employed workers has reported no significant effects of vaccination on mental health^9^. However, the vaccination rate was very low (11%) in its study population, and most of the vaccinated individuals had received one dose of the vaccine within only a week before the survey through the workplace vaccination program.

In a previous cross-sectional study in Japan, it was reported that severe psychological distress was associated with COVID-19 vaccine hesitancy^10^. In an attempt to control for the effects of baseline mental health status on the vaccination behavior, the present study was adjusted for baseline K6 scores and vaccine hesitancy. Moreover, sensitivity analyses excluding people with higher levels of psychological distress (K6 scores ≥ 9 or ≥ 13) at baseline or those who had depression or other mental diseases at baseline (data not shown) were performed and yielded similar results.

There is evidence showing that COVID-19 patients are at increased risk of developing various mental health disorders, including anxiety disorders, depressive disorders, and stress and adjustment disorders, within one year after the acute phase of the infection^11^. Since the risk of SARS-CoV-2 infection is different among people with different vaccination status, the present analyses were adjusted for COVID-19 infection in the past year, which did not change the results.

It could be speculated that vaccine receipt may have indirect effects on mental health in addition to directly lowering psychological distress. For example, vaccinated participants might feel less anxious about being infected or less likely to develop severe COVID-19, and become more socially active, resulting in a positive impact on their mental well-being^12^. However, such indirect effects of vaccination were not evident in the present multivariable or stratified analyses.

The present study has several limitations that must be considered. First, although the associations between vaccination and mental well-being shown in our study were adjusted for baseline K6 scores, a possible reverse causality that mental health status had affected vaccination behavior may not be completely ruled out. However, analyses that excluded people with serious psychological distress at baseline or that adjusted or stratified by baseline vaccine hesitancy did not change the results. Second, we asked participants about their vaccination status in September–October 2021 but we did not obtain data on the date they received the first or second dose of the vaccine. Thus, there were differences in the period from baseline to vaccination or from the vaccination to the follow-up. Difference in the durations might have had some effect on improvement or deterioration of participants’ mental health status. Third, we excluded participants with invalid responses from the study at the beginning using an attention check question. We also considered those who provided extremely unnatural and probably unreal responses to question items about drug usage and existing diseases as invalid, which would have made a robust sample; however, we did not check all discrepant responses through the entire questionnaire. Fourth, the information on mental health status assessed using the PHQ scale was not available for the present study, which has prevented us from direct comparison of findings with the previous studies conducted in the United States and China on the similar topic. However, the K6 scale used in our study includes similar question items to the PHQ such as nervousness, hopelessness, and worthlessness; moreover, it was reported that the K6 scale demonstrated large correlations with the PHQ^13^. Last, the results of the present study may not be generalizable to the Japanese population, especially those who didn’t have access to the survey platform through the internet. However, the JACSIS study was based on a large survey platform with approximately 2.2 million registered members, and it implemented a simple random sampling procedure according to age, sex, and residential prefecture strata to be representative of the Japanese population.

In conclusion, the present study revealed that receiving one or two doses of COVID-19 vaccines might have contributed to the improvement of mental well-being in addition to the vaccine’s effect on the virus control. The bonus advantage of vaccination might provide evidence for the authorities to promote vaccination against COVID-19 as well as emerging infectious diseases in the future.

## Methods

### Participants

The baseline survey was conducted in February 2021, which was before the initiation of the nationwide COVID-19 vaccination program in Japan. The participants of the present study were those who responded to the baseline survey and to the follow-up survey conducted from September to October 2021. The surveys were carried out online through a major Japanese Internet research company (Rakuten Insight, Inc., Tokyo, Japan). The details of the surveys are described elsewhere^14^. Briefly, the baseline and the follow-up surveys of the present study correspond to the second and third wave surveys of the Japan COVID-19 and Society Internet Survey (JACSIS) study, respectively. The invitation to the second wave JACSIS was sent primarily to the participants of the first wave JACSIS, which was carried out from August to September 2020. In order to reach a designated sample size, the invitation was also sent to previous participants of the Japan Society and New Tobacco Internet Survey (JASTIS) ^15^ since the sample selection of the first wave JACSIS and JASTIS were similar. Namely, these surveys recruited participants that would represent the Japanese population regarding sex, age composition from 15 to 79 years, and 47 residential prefectures in Japan using a simple random sampling procedure in approximately 2.2 million registered panelists. In total, 22,326 participated in both baseline (the second wave JACSIS) and the follow-up (the third wave JACSIS) surveys were used for the present study. Those who agreed to participate in each wave of surveys were asked to provide online informed consent before responding to the questionnaires. The study protocol was approved by the ethics committees at Nagoya University School of Medicine (26 August, 2022; approval no. 2022–0185) and Osaka International Cancer Institute (19 June, 2020; approval no. 20084), Japan. We conducted this study in compliance with the 1964 Declaration of Helsinki and its later amendments or comparable ethical standards.

### Exclusion criteria

First, participant who did not pay attention to answer options or just speed through the questionnaire and provided invalid responses were excluded (n = 5114). Namely, (1) those who did not provide a correct response to an attention check question included in the middle of the questionnaire asking people to “select the penultimate one from the following five options,” (2) those who provided “yes” responses to the use of all the nine categories of drugs (morphine, marijuana, cocaine, heroin, etc.), or (3) those who provided “yes” responses to all the 20 health conditions (hypertension, diabetes, asthma, atopic dermatitis, angina, myocardial infarction, stroke, chronic obstructive pulmonary disease, chronic kidney disease, cancers, chronic pain, depression, etc.) were excluded. Second, participants with non-standard vaccination status (n = 123, described later) were excluded, leaving 17,089 for statistical analyses. Due to the nature of the online survey, there were no missing values.

### Variables

#### Mental well-being

Participants’ mental health status was assessed using the Japanese version of the Kessler Psychological Distress Scale (K6), a validated and globally used six-item scale for nonspecific psychological distress with a score range of 0–24 points^16,17^. The recommended cutoffs of 5, 9, and 13 points were used to define mild, moderate, and serious psychological distress, respectively^17,18^. Accordingly, participants who scored 5 points or higher were defined as having psychological distress, those who scored 9 points or higher were defined as having moderate or serious psychological distress, and those who scored 13 points or higher were defined as having serious psychological distress.

#### COVID-19 vaccination status and baseline vaccine hesitancy

Participants’ status of COVID-19 vaccination was asked in the third wave of the JACSIS (September–October 2021) with eight options of answers: (1) have already received two doses, (2) have already received one dose and have been waiting for the second dose, (3) have already received one dose, and do not have any plan for the second dose, 4) have already received one dose of a single-dose type vaccine, (5) could not receive vaccines because of underlying conditions, (6) waiting for the first dose, (7) wait and see, and (8) do not want to receive the vaccine. We categorized the respondents into three groups: “(1) received two doses” as two-dose vaccination, “(2) received one dose” as one-dose vaccination, and “(5–8) didn’t receive any” as no vaccination. Those who had already received one dose but rejected the second dose, or those who had received a single-dose type vaccine, which was not authorized at that time in Japan, were excluded from the analyses (n = 123).

Regarding COVID-19 vaccine hesitancy, participants were asked in the second wave of the JACSIS (baseline): “What do you think about vaccination against new coronavirus infections?” Those who answered that “I want to get vaccinated” or “I want to get vaccinated after seeing how it goes” were defined as “not hesitant”, and those who answered “I don’t want to get vaccinated” were defined as “hesitant”.

### Covariates

Educational attainment was classified into five levels (graduated from junior high school, senior high school, 2-year college, university, or graduate school). Living arrangements (living with family members or not) and occupation (healthcare workers or not) were dichotomized. The Fear of Coronaviruss-19 Scale (FCV-19S) ^19,20^ was used to assess the fear of COVID-19 at follow-up, which is a seven-item questionnaire with each item containing a five-point Likert-type scale responses ranging from 1 (strongly disagree) to 5 (strongly agree). Frequency of outings and frequency of voice chatting with people outside the household not only by face-to-face manner but using telephone, video call, etc. were assessed at the follow-up using seven-category frequency responses (none, once/month, 2–3 days/month, once/week, 2–3 days/week, 4–5 days/week, almost every day). These frequency responses were then converted into continuous numbers (0, 1, 2.5, 4.35, 10.88, 19.58, 28.28 days per month) for the analyses^21^. Information on COVID-19 infection in the past one year (yes/no) was also self-reported at the follow-up.

### Statistical analysis

Mean K6 scores of the three vaccination status groups, i.e., one-dose vaccination, two-dose vaccination, and no vaccination, were calculated using general linear models adjusted for baseline K6 scores, sex, age, educational attainment, living arrangements, occupation, vaccine hesitancy at baseline, and scores of the FCV-19S, frequency of outings, frequency of voice chatting, and history of COVID-19 infection at follow-up. Differences in mean K6 scores among the three groups were tested using the analysis of covariance (ANCOVA). We also conducted analyses excluding subjects with higher psychological distress at baseline (K6 ≥ 9 or ≥ 13).

In an attempt to analytically make a possible causal inference from COVID-19 vaccination to mental health status, we designed two analyses: (1) improvement of mental health and (2) deterioration of mental health from the baseline to the follow-up survey according to different vaccination statuses in September to October 2021. For the former (1) analysis examining the association between COVID-19 vaccination and the improvement in mental health, participants who had psychological distress (K6 ≥ 5) at baseline (n = 6583) were selected. Improvement was defined as follow-up K6 < 5. In contrast, for the latter (2) analysis examining the association between COVID-19 vaccination and the deterioration in mental health, those who did not have psychological distress (K6 < 5) at baseline (n = 10,506) were selected for the analysis. Deterioration was defined as follow-up K6 ≥ 5. Multivariable logistic regression models adjusted for baseline K6 scores and other covariates described above were used and odds ratios (ORs) and 95% confidence intervals (CIs) of improvement or deterioration of mental health statuses derived from the models are reported. We also conducted the same analyses by adopting different K6 cutoffs for the outcome variable as sensitivity analyses: 9 points (≥ 9 vs. < 9) or 13 points (≥ 13 vs. < 13).

Stratified analyses by three age groups (15–39, 40–64, 65 years and over), vaccine hesitancy at baseline, fear of COVID-19 (FCV-19S < 19 and ≥ 19 points) at follow-up, and outing frequency (< 3 and ≥ 3 days/week) at follow-up were also performed as supplementary analyses. All statistical tests in the present study were two-tailed and statistical significance was set at p values < 0.05. All statistical analyses were performed using IBM SPSS Statistics for Windows, Version 28.0 (Armonk, NY: IBM Corp).

## Supplementary Information

Supplementary Information.

## Data Availability

All anonymized individual participant data reported in this paper are available for interested researchers who send a request for data sharing, along with a synopsis of the secondary analysis plan paper to the last author (T.T.).
