# Supplementary material for: Possible contribution of COVID-19 vaccination to the subsequent mental well-being in Japan
Source: Sci Rep. 2022 Dec 7;12:21195. doi: 10.1038/s41598-022-25357-1 (PMC9729563; doi:10.1038/s41598-022-25357-1)
Supplement: Supplementary file 1 — Supplementary Information. [file 41598_2022_25357_MOESM1_ESM.pdf]

Possible contribution of COVID-19 vaccination to the subsequent mental well-being in Japan

Chifa Chiang, Shuhei Morita, Yoshihisa Hirakawa, Farzana Tanzin Priya, Yuka Matsumoto, Atsuhiko Ota, Hiroshi Yatsuya, Takahiro Tabuchi

Supplementary Table 1. Mean K6 scores in participants without higher levels of baseline psychological distress according to COVID-19 vaccination status, 2021, JACSIS, Japan

	No vaccination	One-dose vaccination	Two-dose vaccination
Participants with a baseline K6 score equal to 0-8 (n=13242)			
n	2062	983	10197
Mean K6 at baseline (SE)			
crude	2.22 (0.06)	2.50 (0.08)	2.04 (0.02)
adjusted, Model 1	2.04 (0.06)	2.23 (0.08)	2.10 (0.02)
Mean K6 at follow-up (SE)			
crude	3.05 (0.09)	2.80 (0.13)	2.13 (0.03)
adjusted, Model 1	2.66 (0.08)	2.25 (0.12)	2.26 (0.04)
adjusted, Model 2	2.66 (0.08)	2.18 (0.10)	2.26 (0.03)
Participants with a baseline K6 score equal to 0-12 (n=15326)			
n	2508	1185	11633
Mean K6 at baseline (SE)			
crude	3.77 (0.08)	3.90 (0.11)	3.12 (0.03)
adjusted, Model 1	3.32 (0.08)	3.30 (0.11)	3.27 (0.03)
Mean K6 at follow-up (SE)			
crude	4.06 (0.10)	3.59 (0.13)	2.77 (0.04)
adjusted, Model 1	3.53 (0.08)	2.87 (0.12)	2.96 (0.04)
adjusted, Model 2	3.49 (0.08)	2.88 (0.10)	2.97 (0.03)

K6: Kessler 6 scale; COVID-19: coronavirus disease 2019; SE: standard error

Model 1: adjusted for sex, age, education, occupation, living arrangements

Model 2: adjusted for sex, age, education, occupation, living arrangements, baseline K6, baseline vaccine hesitancy, frequency of outings at follow-up, frequency of voice chatting at follow-up, fear of COVID-19 scores at follow-up, and COVID-19 infection

Supplementary Table 2. Adjusted odds ratios of improved mental health according to COVID-19 vaccination status in participants with psychological distress at baseline, 2021, JACSIS, Japan

	No vaccination	One-dose vaccination	Two-dose vaccination
Total (n=6583)			
n	1357	682	4544
Improved %	27.2	32.3	37.5
aOR (95% CI), Model 1	reference	1.31 (1.07-1.61)	1.38 (1.20-1.59)
aOR (95% CI), Model 2	reference	1.32 (1.06-1.64)	1.35 (1.16-1.58)
aOR (95% CI), Model 3	reference	1.31 (1.05-1.63)	1.35 (1.15-1.57)
Stratified by age			
15-39 years (n=2190)			
n	636	342	1212
Improved %	25.3	28.4	32.5
aOR (95% CI), Model 3	reference	1.10 (0.80-1.51)	1.39 (1.01-1.77)
40-64 years (n=3335)			
n	639	330	2366
Improved %	28.2	36.1	34.0
aOR (95% CI), Model 3	reference	1.58 (1.16-2.17)	1.25 (1.00-1.56)
65 years or over (n=1058)			
n	82	10	966
Improved %	34.1	40.0	52.4
aOR (95% CI), Model 3	reference	1.15 (0.26-5.07)	1.92 (1.08-3.44)
Stratified by vaccine hesitancy at baseline			
Not hesitant (n=5759)			
n	913	599	4247
Improved %	26.3	33.2	37.8
aOR (95% CI), Model 3[a]	reference	1.38 (1.09-1.76)	1.38 (1.15-1.64)
Hesitant (n=824)			
n	444	83	297
Improved %	29.1	25.3	33.0
aOR (95% CI), Model 3[a]	reference	0.96 (0.53-1.73)	1.30 (0.91-1.85)
Stratified by fear of COVID-19 at follow-up			
FCV-19S <19 (n=2519)			
n	575	298	1646

Improved %	34.4	38.3	42.3
aOR (95% CI), Model 3[a]	reference	1.30 (0.93-1.80)	1.13 (0.89-1.43)
FCV-19S >=19 (n=4064)			
n	782	384	2898
Improved %	21.9	27.6	34.8
aOR (95% CI), Model 3[a]	reference	1.36 (1.01-1.83)	1.56 (1.27-1.93)
Stratified by outing frequency at follow-up			
Outings <3 days/week (n=3851)			
n	839	398	2614
Improved %	24.9	31.2	35.3
aOR (95% CI), Model 3[a]	reference	1.32 (0.99-1.77)	1.40 (1.14-1.71)
Outings >=3 days/week (n=2732)			
n	518	284	1930
Improved %	30.9	33.8	40.5
aOR (95% CI), Model 3[a]	reference	1.28 (0.92-1.79)	1.29 (1.02-1.64)

COVID-19: coronavirus disease 2019; aOR: adjusted odds ratio; CI: confidence interval; FCV-19S: Fear of COVID-19 scale; K6: Kessler 6 scale

Model 1: adjusted for sex, age, education, occupation, living arrangements

Model 2: adjusted for variables in Model 1 plus baseline K6 and baseline vaccine hesitancy

Model 3: adjusted for variables in Model 2 plus frequency of outings at follow-up, frequency of voice chatting at follow-up, FCV-19S score at follow-up, and COVID-19 infection

[a] Variable for stratification is not included in the model

Supplementary Table 3. Adjusted odds ratios of deteriorated mental health according to COVID-19 vaccination status in participants without psychological distress at baseline, 2021, JACSIS, Japan

	No vaccination	One-dose vaccination	Two-dose vaccination
Total (n=10506)			
n	1585	735	8186
Deteriorated %	19.6	16.1	11.4
aOR (95% CI), Model 1	reference	0.71 (0.56-0.90)	0.71 (0.61-0.83)
aOR (95% CI), Model 2	reference	0.67 (0.53-0.86)	0.71 (0.60-0.84)
aOR (95% CI), Model 3	reference	0.66 (0.51-0.85)	0.70 (0.59-0.83)
Stratified by age			
15-39 years (n=1806)			
n	506	304	996
Deteriorated %	27.3	17.1	21.7
aOR (95% CI), Model 3	reference	0.58 (0.40-0.86)	0.75 (0.57-0.99)
40-64 years (n=4789)			
n	815	406	3568
Deteriorated %	17.5	15.3	12.4
aOR (95% CI), Model 3	reference	0.74 (0.52-1.06)	0.77 (0.60-0.97)
65 years and over (n=3911)			
n	264	25	3622
Deteriorated %	11.4	16.0	7.6
aOR (95% CI), Model 3	reference	0.98 (0.28-3.44)	0.48 (0.30-0.78)
Stratified by vaccine hesitancy at baseline			
Not hesitant (n=9407)			
n	997	641	7769
Deteriorated %	20.1	16.1	11.2
aOR (95% CI), Model 3[a]	reference	0.68 (0.51-0.90)	0.68 (0.56-0.83)
Hesitant (n=1099)			
n	588	94	417
Deteriorated %	18.9	16.0	14.9
aOR (95% CI), Model 3[a]	reference	0.55 (0.29-1.05)	0.81 (0.56-1.17)
Stratified by fear of COVID-19 at follow-up			
FCV-19S <19 (n=5774)			
n	939	420	4415

Deteriorated %	13.2	10.7	6.7
aOR (95% CI), Model 3[a]	reference	0.69 (0.47-1.01)	0.63 (0.49-0.82)
FCV-19S >=19 (n=4732)			
n	646	315	3771
Deteriorated %	28.9	23.2	17.0
aOR (95% CI), Model 3[a]	reference	0.68 (0.48-0.94)	0.78 (0.62-0.97)
Stratified by outing frequency at follow-up			
Outings <3 days/week (n=5281)			
n	902	392	3987
Deteriorated %	21.5	20.7	12.8
aOR (95% CI), Model 3[a]	reference	0.80 (0.58-1.10)	0.73 (0.59-0.91)
Outings >=3 days/week (n=5225)			
n	683	343	4199
Deteriorated %	17.1	10.8	10.1
aOR (95% CI), Model 3[a]	reference	0.46 (0.30-0.71)	0.64 (0.49-0.84)

COVID-19: coronavirus disease 2019; aOR: adjusted odds ratio; CI: confidence interval; FCV-19S: Fear of COVID-19 scale; K6: Kessler 6 scale

Model 1: adjusted for sex, age, education, occupation, living arrangements

Model 2: adjusted for variables in Model 1 plus baseline K6 and baseline vaccine hesitancy

Model 3: adjusted for variables in Model 2 plus frequency of outings at follow-up, frequency of voice chatting at follow-up, FCV-19S score at follow-up, and COVID-19 infection

[a] Variable for stratification is not included in the model
